# Protocol for a systematic review and meta-analysis of coordinate-based network mapping of brain structure in bipolar disorder across the lifespan

**DOI:** 10.1192/bjo.2023.569

**Published:** 2023-10-09

**Authors:** Brett D. M. Jones, Peter Zhukovsky, Colin Hawco, Abigail Ortiz, Andrea Cipriani, Aristotle N. Voineskos, Benoit H. Mulsant, Muhammad Ishrat Husain

**Affiliations:** Campbell Family Mental Health Research Institute, Centre for Addiction and Mental Health, Canada; and Department of Psychiatry, Temerty Faculty of Medicine, University of Toronto, Canada; Department of Psychiatry, University of Oxford, UK; Oxford Health NHS Foundation Trust, Warneford Hospital, Oxford, UK; and Oxford Precision Psychiatry Laboratory, NIHR Oxford Health Biomedical Research Centre, UK

**Keywords:** Bipolar disorders, other imaging, depressive disorders, review, connectivity

## Abstract

**Background:**

Studies about brain structure in bipolar disorder have reported conflicting findings. These findings may be explained by the high degree of heterogeneity within bipolar disorder, especially if structural differences are mapped to single brain regions rather than networks.

**Aims:**

We aim to complete a systematic review and meta-analysis to identify brain networks underlying structural abnormalities observed on T1-weighted magnetic resonance imaging scans in bipolar disorder across the lifespan. We also aim to explore how these brain networks are affected by sociodemographic and clinical heterogeneity in bipolar disorder.

**Method:**

We will include case–control studies that focus on whole-brain analyses of structural differences between participants of any age with a standardised diagnosis of bipolar disorder and controls. The electronic databases Medline, PsycINFO and Web of Science will be searched. We will complete an activation likelihood estimation analysis and a novel coordinate-based network mapping approach to identify specific brain regions and brain circuits affected in bipolar disorder or relevant subgroups. Meta-regressions will examine the effect of sociodemographic and clinical variables on identified brain circuits.

**Conclusions:**

Findings from this systematic review and meta-analysis will enhance understanding of the pathophysiology of bipolar disorder. The results will identify brain circuitry implicated in bipolar disorder, and how they may relate to relevant sociodemographic and clinical variables across the lifespan.

## Background

Bipolar disorder is a major cause of global burden with significant morbidity and mortality.^1^ It is a heterogenous disorder with onset across the lifespan and various trajectories. Over the course of illness, patients present with a combination of distinct mood states, including major depressive episodes, hypomania, mania, euthymia and mixed episodes. In addition, there is a high degree of associated symptoms, such as cognitive impairment, and psychiatric or physical comorbidity.^2–5^ There is also evidence suggesting that bipolar disorder is a neuroprogressive illness that presents differently across the lifespan, further contributing to overall heterogeneity.^5,6^ There is evidence to suggest that bipolar disorder impairs adolescent brain development and accelerates structural brain decline in adults.^7^ One explanation for these varying presentations could be that recurrent or chronic mood episodes combined with lifestyle factors lead to pathological processes that contribute to cumulative neural damage and the progressive nature of bipolar disorder.^6^ Consequently, a nuanced understanding of the neural pathophysiology of bipolar disorder at different ages and how it relates to specific clinical profiles could advance the development of better targeted acute and maintenance treatments.

There have been several attempts at determining brain abnormalities underlying bipolar disorder by using structural neuroimaging studies; however, findings have been inconsistent.^8^ In a large meta-analysis, the most replicated differences between patients with bipolar disorder and controls included reduced thickness in the left anterior cingulate, left paracingulate, left superior temporal gyrus and prefrontal regions bilaterally.^8^ The ENIGMA Bipolar Disorder Working Group subsequently completed a large analysis of cortical grey matter thickness in persons with bipolar disorder. They found that grey matter was thinner across most of the cortex of both brain hemispheres; most strongly in the left pars opercularis, left fusiform gyrus and left rostral middle frontal cortex.^9^ Few studies have focused on late-life bipolar disorder or analysis of longitudinal cohorts of patients with bipolar disorder. In a recent longitudinal analysis of structural magnetic resonance imaging (MRI) data, patients with bipolar disorder had faster enlargement of ventricular volumes as they aged than controls.^10^ In addition, more hypomanic or manic episodes were associated with accelerated cortical thinning, primarily in the prefrontal cortex.^10^ Research into late-life bipolar disorder has suggested that a subset of patients show more pronounced grey and white matter declines with age.^11^ Despite the large body of research, findings remain inconsistent. These inconsistencies could be because of genetic and clinical heterogeneity in bipolar disorder across samples (e.g. mean age, duration of illness, predominant lifetime polarity, medications, psychiatric or physical comorbidity).^12,13^ Alternatively, different patterns may emerge among subgroups of patients with bipolar disorder, representing different neurobiological pathways.^14,15^ Also, neuropsychiatric symptoms and disorders may be better localised to brain networks rather than single brain regions.^16,17^ However, most available studies (and their meta-analyses) have focused on single brain regions.

Coordinate-based network mapping (CBNM) is a novel meta-analytic approach for neuroimaging studies to assess brain networks rather than individual regions. This approach uses the normative human brain connectome to establish significant differences in networks of connectivity between patient and control populations. This approach uses the normative human brain connectome to map case–control differences in brain structure to functional networks, and thus allows us to test differences in networks affected by the psychiatric condition. First, locations of case–control differences are leveraged to network maps identifying braid regions functionally connected to these locations in a large sample of healthy adults. Next, the resulting network maps are analysed in meta regressions and compared across studies. It has been used to map psychiatric symptoms and clinical profiles in disorders such as Parkinson's disease and major depressive disorder.^18,19^

## Objective

In this protocol, we describe the methods of a systematic review and meta-analysis utilising both activation likelihood estimate (ALE) and novel CBNM in bipolar disorder. Our aims are (a) to identify the brain networks underlying structural abnormalities observed on T1-weighted MRI scans in bipolar disorder across the lifespan, and (b) to explore how these brain networks are affected by sociodemographic and clinical heterogeneity in bipolar disorder.

## Method

This protocol is reported in accordance with the Preferred Reporting Items for Systematic Reviews and Meta-analyses Statement^20^ and is registered with the International Prospective Register of Systematic Reviews (PROSPERO; identifier CRD42023413964).

### Eligibility criteria

We will include studies that focus on whole-brain analyses of structural differences between controls and participants of any age, with a diagnosis of bipolar disorder according to the criteria of the DSM-III, ICD-10 or the Chinese Classification and Diagnosis Criteria of Mental Disorder (CCMD-1).^21–23^ We will include studies that recruited a variety of participants, including those with psychiatric or physical comorbidity, those who are medication naive or receiving medication, and those in any polarity (i.e. euthymia; mixed, depressive or (hypo)manic episode). However, we will exclude studies that explicitly recruited participants with comorbid disorders that directly affect brain structure, such as stroke, neurodegenerative disorders, traumatic brain injury, autoimmune or inflammatory demyelinating disease, or cancer. Transdiagnostic samples (i.e. participants with various psychiatric diagnoses) will be included if participants with bipolar disorder are reported separately or if they constitute more than 85% of the sample. We will not include studies without a non-psychiatric control group.

We will include cross-sectional, case–control or cohort studies from any setting. We will not include case reports and reviews or meta-analyses (e.g. ENIGMA Consortium). We will include both English and non-English studies published from inception to present date.

We will include studies that focused on whole-brain analysis of structural differences, using voxel-based morphometry or surface-based cortical thickness or volume analysis. We will exclude studies that (a) focus on regions of interest rather than the whole brain, (b) are solely correlational analyses, (c) did not report any differences between patients and control participants (CBNM currently cannot synthesize null results), (d) focused on non-grey matter or (e) did not report coordinates or a specific region of interest to be extracted. We will attempt to contact authors of papers that only reported regions of interest where it is suspected that whole-brain data may be available or to obtain relevant coordinates.

### Information sources

The electronic databases Medline, PsycINFO and Web of Science will be searched. Key terms, notable papers and citation lists will also be reviewed for additional studies.

### Search strategy

The following is a draft search strategy from Medline, adapted from previous work:^19^

(exp Bipolar Disorder/ or Bipolar.mp. OR Bipolar Depression.mp. OR Bipolar*.mp. OR Manic Depres*.mp. OR affective psychosis) AND (((voxel-based morphometry or voxel-based or VBM or cortical thickness).mp OR voxel-bas* morphometri* or cortic* region* or cortic* thick* or brain* structur* or brain* volum* or structur* brain* network* or structur* covari* network* or cerebellar* volum* or cortic* thin* or cortic* volum* or high-resolut* structur* or surface-bas* morphometri* or structur* differ* or volum* differ* or cortic* surfac* or structur* brain* correl* or volum* increas* or brain* morpholog* or cortic* morpholog* or brain* morphometri* or ‘comput* anatomi* toolbox* OR t1-weight* structur* magnet* reson* imag* OR cortic* gyrif* OR surface OR volum* reduct* OR volumetr* reduct* OR multi-mod* magnet* reson* imag* OR brain* structur* integr*’ or voxel-bas* lesion-symptom* mapping or gray-matt* volum* or voxel* base* morphometri* or smaller* hippocamp* volum* or subcort* pattern* or hippocamp* subfield* volum* or matter* volum* chang* or grey-matt* volum* or reduc* hippocamp* volum* or brain* atrophi*)).

### Study records

#### Data management

Articles will be uploaded to and managed in the Covidence software for Windows (Covidence systematic review software, Veritas Health Innovation, Melbourne, Australia; see www.covidence.org). Extracted data will be stored in a shared Microsoft Excel (version 16.67 for Windows) file within the study group.

#### Selection process

Two authors will independently screen the titles and abstracts, based upon predefined screening criteria. Full texts of relevant studies will be reviewed by two independent reviewers, based upon the same inclusion criteria.

#### Data extraction and data items

Data will be extracted by two independent authors, using a predetermined extraction template. Any discrepancies of inclusion or extraction will be discussed between the two authors, and a third author will resolve any further conflicts.

Data will be extracted broadly based upon the following categories: study characteristics (e.g. publication year, region, funding source), patient sociodemographic characteristics (e.g. age, gender distribution, education) and clinical variables (e.g. bipolar type, mood state, severity of depressive or manic symptoms, suicidality, cognitive performance, age at onset and duration of illness, history of psychosis, psychiatric comorbidity, physical comorbidity, psychotropic and non-psychotropic medications).

### Outcomes

The primary outcome will be whole-brain coordinate comparisons of grey matter integrity and structure, including voxel-based morphometry of grey matter density and volume (typically conducted in FMRIB Software Library (FSL) or Statistical Parametric Mapping (SPM)) and surface-based morphometry measures of cortical thickness (typically obtained with the software FreeSurfer).

### Quality of data and risk of bias

A modified version of the Newcastle–Ottawa Scale for cross-sectional studies will be used to assess the quality of the included studies.^24^ For observational studies, we will use the Risk of Bias in Non-Randomized Studies – of Exposures (ROBINS-E) tool to measure risk of bias.^25^ To assess publication bias, publications will also be evaluated with funnel plot asymmetry and Egger's regression test on effect size estimates extracted from significant clusters.^26^

### Data synthesis

We will carry out a meta-analysis to synthesise the data about structural differences between cases and controls identified in each study.

The primary analysis will be a CBNM meta-analytic approach.^18,27^ We will identify study-specific seed maps that locate the differences between cases and controls, and will then use CBNM to generate study-specific network maps.^13^ Using seed-based functional connectivity analyses (dual_regression tool in FSL) in unrelated Human Connectome Project participants, we will use study-specific coordinates or regions of interest to study-specific network maps.^28–30^ We will then threshold the network maps across studies to generate group mean maps. Using meta-regressions on the network maps, we will assess the effects of relevant study characteristics, patient demographics and clinical variables from the extracted data on the study-specific networks.

To make the current review comparable to previous work, we will also report an ALE meta-analyses conducted with the GingerALE (version 3.0.2 for Windows; Brainmap, San Antonio, Texas, USA; see brainmap.org/ale) software program, based on the same coordinates as those used in the lesion-network approach described above. The combination of these two meta-analytic approaches will allow readers to assess how the CBNM compares with the standard meta-analytic approach.

### Subgroup analyses

We will perform several subgroup analyses. We will group patients based upon sociodemographic and clinical variables such as late-life bipolar disorder (e.g. those with a mean age ≥50 years), younger-adult bipolar disorder (with a mean age ≤49 and >17 years), paediatric bipolar disorder (PBD; with a mean age ≤17 years), psychiatric comorbidity, physical comorbidity, medication naïve or receiving treatment, current medications (e.g. lithium, antipsychotics), mood state, history of psychosis, duration of illness and early (≤49 years) or late (≥50 years) age at onset. We will repeat the primary analysis to assess networks involved in these subgroups and how they may differ.

### Planned sensitivity analyses, cross-validation and assessment of heterogeneity

Reliability of the results will be examined by sensitivity analysis of relevant variables. As we suspect there may be limited reports for specific groups (e.g. PBD), we will also repeat the subgroup analyses with more flexible criteria (e.g. increasing the age range of PBD to include those aged up to 25 years). We will also conduct a jackknife analysis, which consists of repeating the main analysis as many times as studies include, removing one different study each repetition. Heterogeneity will be assessed with the *I*^2^-statistic.

### Confidence of cumulative evidence

A Grading of Recommendations, Assessment, Development and Evaluations (GRADE) assessment will be used to evaluate the quality of the cumulative evidence.

## Discussion

Bipolar disorder is a chronic illness that occurs across the lifespan. It is a heterogenous disorder with significant psychiatric and physical comorbidity.^2–4^ Much work has been done to investigate structural brain abnormalities to understand the pathophysiology of bipolar disorder, focused largely on lesion analyses.^9,10^ We plan to conduct a systematic review utilising a novel meta-analytic approach, CBNM, to determine network abnormalities in bipolar disorder by using data from the human connectome. The most common coordinate-based meta-analytic method is ALE, which aims to determine the convergence of activation probabilities between experiments.^31^ The results represent convergence in specific brain regions of all included studies that is higher than chance.^31^ Although previous studies have reported significant convergence of activation probabilities in bipolar disorder, coordinate convergence on single brain regions may not fully explain structural abnormalities in all presentation of bipolar disorder.^32^ To expand conventional coordinate-based meta-analyses, CBNM has been used to map networks for specific psychiatric symptoms of individual psychiatric disorders and to determine common networks across several psychiatric disorders.^18,33–35^ Using both CBNM and ALE in our analyses of bipolar disorder may improve our understanding of this highly heterogenous disorder, as well as allow us to compare the results of CBNM and an updated gold-standard analysis (i.e. ALE). This approach still has some limitations, as we will only synthesize significant findings and will not consider direction of the case–control differences. CBNM may also introduce biases inherent to connectivity profiles. Identified regions and nodes in the network may not have a causal role in the disorder or its symptoms. Finally, a limitation of the planned analysis may be that there are fewer reports from the older and younger populations. We will address this limitation by conducting subgroup analysis with the age groups, as well as assessing age as a linear variable. Overall, this work may provide a more nuanced understanding of brain abnormalities in bipolar disorder, and how they relate to sociodemographic and clinical heterogeneity. It will integrate both single brain region and networks implicated in bipolar disorder, providing an opportunity for the development of more targeted treatments for patients with specific phenotypic presentations.

## Data Availability

Data availability is not applicable to this article as no new data were created or analysed in this study.
